# Implementation of a modified drive-through sampling strategy for SARS-CoV-2-the Nigerian experience

**DOI:** 10.11604/pamj.supp.2020.35.2.24319

**Published:** 2020-07-09

**Authors:** Olufemi Samuel Amoo, Aigbe Gregory Ohihoin, Adesola Zaidat Musa, Ifeoma Idighe, Fehintola Ige, Temie Giwa-Tubosun, Sodiq Oloko, Aisha Abiola, Esther Ngozi Ohihoin, Agatha Ngozi David, Abideen Salako, David Oladele, Chidinma Muoghalu Gab-Okafor, Tajudeen Akanji Bamidele, Oluwagbemiga Olanrewaju Aina, Emelda Chukwu, Nkiruka Nnonyelum Odunukwe, Oliver Chukwujekwu Ezechi, Rosemary Ajuma Audu, Babatunde Lawal Salako

**Affiliations:** 1 Nigerian Institute of Medical Research 6 Edmund Crescent, Yaba, Lagos, Nigeria,; 2 Life Bank, Lagos, Nigeria

**Keywords:** COVID-19, SARS–CoV-2, drive-through testing, coronavirus disease

## Abstract

**Introduction:**

effective and safe means of sample collection is a crucial component of testing for Covid-19. Uptake of testing is key to containing and controlling the spread of the virus. Scientists have been working on various strategies that will increase the uptake of testing for COVID-19. One such method involves the use of the drive-through sampling strategy.

**Methods:**

data was collected by both qualitative and quantitative methods. An eligibility form was filled online. While in-depth interviews were conducted for the qualitative aspect of the study.

**Results:**

2,600 visits were recorded at the website, 2300 (88.46%) participants successfully registered for the test. 57.4% were found eligible of which 78.0% presented for the test. This Consisted of 78.0% drive-through and 22.0% walk-in. The average time for transiting through the drive-through site was 19.2 ± 4.6minutes while that of the walk-in was 28 ± 9.2min. This difference was statistically significant (p<0.001). In the qualitative component, respondents opined that maximum safety measures were deployed to protect both participants and health workers. Most said that the turnaround time for the sampling process was short.

**Conclusion:**

the sampling strategy although largely successful, is largely dependent on Internet penetrability, thus this sampling modality will be best utilized as an adjunct to established models of sample collection.

## Introduction

The novel coronavirus disease (COVID-19) was declared a global pandemic by the WHO on 11th March 2020 [1]. Since then, there has been a global effort to ramp up strategies to contain and defeat the pandemic. Crucial to this global effort is the place of scaling up testing services to identify those who are already infected, track their contacts, get them tested as well as commence treatment to contain the spread of the virus [2]. Effective and safe means of sample collection is a crucial component of testing. Before the pandemic, the conventional modality for sample collection involves a special laboratory space within the health facility designated for conducting the test and an extensive process to ensure the decontamination of the space, materials, and personnel involved [3]. This measure is to prevent health personnel involved in the procedure from being unduly exposed to the risk of infection. Due to these bottlenecks, the uptake of the test in terms of the number of patients sampled becomes limited. Considering that the uptake of testing is key to containing and controlling the spread of the virus, scientists have been working on various strategies that will increase the uptake of testing for COVID-19 and also maximize the use of resources while reducing the risk of transmission of the virus to healthcare personnel [4,5]. One such method involves the use of the drive-through sampling strategy [4]. This strategy was first deployed in testing for COVID-19 in South Korea with great success [6]. The use of this method of sampling was found to be effective in early detection and diagnosis of cases and invariably greater ease of contact tracing. This method also comes with the added advantage of reduced risk of infection transmission and less requirement for resources such as PPEs [6]. In the United States of America, this method has been replicated in various centres with similar results as documented in South Korea [4].

The goal of this modality of sample collection is to minimize the risk of transmission of infection to the health care workers, while ensuring efficient use of resources. In the drive-through strategy of sampling, the patients being sampled drive through strategic points in the sample collection centre that are well demarcated and labelled to ensure minimal or no contact with the health personnel involved with sample collection. Samples are collected from the participant without the individual coming out of the car. The only time any health personnel comes in contact with the participant is during the collection of the nasal and oropharyngeal swab. Nigeria is the most populous nation in Africa with approximately 200 million people, mostly living in clusters [7]. Lagos, the former administrative and current commercial capital of the nation with over 20 million people clustered in a small landmass, has a population density of 6,871 persons per sq kilometre [8]. This is more than thirty times the average population density of the country. Lagos has consistently been home to the majority of the confirmed cases diagnosed in the country [9]. Sample collection for testing for Covid-19 in Lagos is therefore a significant challenge. There is, therefore, an urgent need to evaluate other innovative methods of sample collection for Covid-19 to be deployed across the nation. The drive-through modality, although shown to be effective in high-income countries, may not attain the same level of success in low resource settings where personal ownership of cars may be a luxury. It is, therefore, necessary to modify the existing drive-through sampling strategy to accommodate persons who may walk into the test centres. On March 30, 2020, the Nigerian Institute of Medical Research (NIMR) established the first modified drive-through centre for COVID-19 in the West African sub-region by introducing a walk-in component to the drive-through system. This study, therefore, aims to evaluate the successes and challenges of implementing a modified drive-through system for SARS-CoV-2 in Nigeria. Information obtained will inform policy for the scale-up of testing services in the country.

## Methods

**Study Setting:** the Nigerian Institute of Medical Research (NIMR), the apex medical research institute in the country is an agency of the Federal Ministry of Health (FMOH) and one of the designated centers for SARS-CoV-2 testing. NIMR is located in Lagos Mainland and easily accessible from all parts of the state. Before the implementation of the modified drive-through strategy, testing for SARS-CoV-2 was done at the Institute´s Center for Human Virology and Genomics (CHVG) including sample collection and laboratory analysis.

**Study design and duration:** a mixed-method design was used for this study for a duration of 4 weeks. Data was collected by both qualitative and quantitative methods. An eligibility form was filled online and the structured Nigeria Centre for Disease Control (NCDC) “Case Definition Form” was used for the quantitative data collection while in-depth interviews were conducted for the qualitative aspect of the study.

**Figure 1:**
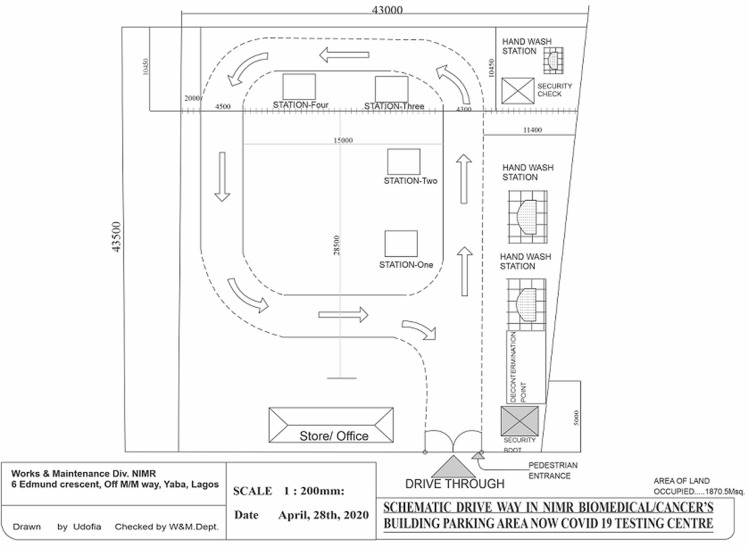
schematic representation of the NIMR drive through

**Procedure:** information on the availability of testing at the center was disseminated through the website and other social media platforms inviting persons with suspicion of COVID-19 infection to register. An online form was completed and the study team reviewed the eligibility of the participants from the back end [8]. Participants also had to indicate whether they would be walk-in or drive-through clients. Text messages or emails were sent to eligible persons inviting them for the test. The messages had a uniquely coded identifier [example- NCV-00111] with a bar code stating the location of the center, the test date and time. Those who did not meet eligibility criteria were given feedback that they were not eligible. For eligible participants, as they drive through or walk to the centre, they were met by security members of the team who granted them access if they showed evidence of invitation. The participants had to go through four designated stations; decontamination, data collection, sampling-material collection point, and point of final collection of samples. At the decontamination station, drive-through participants had their cars decontaminated. At the data collection station, data was collected using the NCDC form (9). At the third station, pre-packed sample collection materials were inserted between the windshield and the wiper. Participants were thereafter directed to the final station for sample collection. Collection involved the use of a special swab stick designed for the collection of samples for SARS-CoV-2. The collected swabs were then transported to the laboratory for analysis (Figure 1).

**Walk-in participants:** at the entrance, the walk-in participants had their invitation verified and were thereafter decontaminated. The participants washed their hands with soap and running water for at least 20 seconds. The soap dispenser and tap were automated to minimize contact and ensure infection control. They then proceeded to the data collection point through a pathway with standpoints which were demarcated two meters apart, to ensure physical distancing. After collection of surveillance and laboratory data, participants were given their sample collection kit and directed to the sample collection station. At this station, both naso- and oropharyngeal samples were collected and packaged as in the drive-through participants. Before exiting the premises, walk-in participants washed their hands and were again decontaminated.

**Personnel:** a total of twenty-seven personnel were involved in the modified drive-through sampling strategy consisting of five environmental officers, six security officers, two infection control officers, four data officers, six laboratory scientists, and four logistic personnel.

**Data collection and analysis:** quantitative data was captured on the NIMR REDCap platform and exported to SPSS for statistical analysis and descriptive statistics used to summarize the data. Qualitative data was collected through phone interviews in the English language. An Interview guide was used to obtain information on the socio-demographic characteristics, and their perception and thoughts on the drive-through centre in NIMR. Salient points around their experiences, safety precautions, and feelings about the Coronavirus test were also explored. The interviews were digitally recorded and transcribed verbatim and analyzed using thematic analysis to provide emerging themes.

## Results

A total of 2,600 visits were recorded at the website, however, only 2300 (88.46%) participants successfully registered for the test. Out of the total number that successfully registered for the test, 57.4% (1320) of participants met the eligibility criteria and were invited for the test. A total of 1030(78.0%) out of the 1320 presented for the drive-through sampling. The 1030 participants consist of 227 (22.0%) walk-in and 803(78.0%) drive-through participants. The time taken to complete the sample collection cycle range from 16 to 34 minutes with a mean of 23.6±6.9 minutes and an interquartile range of 16-35 minutes. The mean time for walk-in sample collection of 28±9.2 minutes is statistically significantly longer than the drive-through sample collection time of 19.17±4.6 minutes (p<0.001). Although no health worker developed symptoms suggestive of COVID-19, 2 (7.4%) out of 27 tested positive for SARS-CoV-2. Of this 27 personnel engaged for the drive-through strategy, 6 were required per testing cycle. The unit cost of conducting the test for one participant ranges from 17.5-21.3 USD with a mean of 19.38 USD. A total of 12 participants were interviewed for the qualitative component of the study; six health workers and six clients. There were eight males and four females. Their ages ranged from 28 to 39 years. All had a minimum of tertiary education and engaged in meaningful means of livelihood. The result of the interviews were arranged in themes following thematic analysis and detailed below.

**Safety measures:** all the respondents interviewed agreed that maximal safety precaution was observed and adhered to. Health workers wore the PPE, clients were provided with masks and sanitizer, their cars were disinfected, drive-through clients were not allowed to come out of their cars and the walk-in observed respiratory and hand hygiene practices. Safety precaution was also observed while transporting samples to the laboratory and electronic dispatch of results. All the clients had adequate knowledge of safety precautionary measures as they could mention and demonstrate how to carry out respiratory and hand hygiene practices. All the health workers reported having adequate PPEs.

**Turn-around time for sample collection and test results:** the time stated by clients for sample collection was between fifteen minutes to thirty-five minutes while the turn-around time to obtain results was between twenty-four hours to 72 hours. A respondent who received the result some days after felt that the delay was probably attributed to a backlog.

**Restriction of movement experience during the COVID-19 outbreak:** all the clients believed that the initiative was good, as it will help to curb the rate of getting infected. The majority of the clients stated that awareness should be created and people educated about the COVID-19 outbreak as people have been walking around in the streets and queuing up to enter supermarkets. Also, they believe that security officials should be educated and trained to know how to respond during outbreaks. Respondent 10 stated, ‘I was stopped by an officer on my way to get tested, I explained that I was going for a test and the officer continued to probe to ask what test I was going for, when I told him it was to test for Corona, he asked me how come?’. I felt he was intruding in my private life. Another officer asked me where I was going to, when I brought out my phone to show him the mail sent inviting me to do the test, he thought I was trying to record him and said I should go, that he did not want any trouble.

**Overall experience with the drive-through centre:** all the clients were impressed and gave a rating of between eighty to ninety-nine point nine per cent. Members of staff were super professional, adhered to safety precaution methods, and were helpful. It was a good initiative from NIMR to test. Respondent 6 stated ‘The NIMR staff are very professional, right from the gate our car was disinfected, we were given face masks, information about the test was provided and the test was done on time’. Another respondent stated, ‘I was scared to take the test but when I came to NIMR, they spoke to me and made me feel comfortable and relaxed, also I spoke to a counsellor who reassured me of the process’ (Respondent 4).

**Walkthrough Experience:** some of the clients who had to come to the centre by public transport stated that the hitches experienced in coming to the centre were: i) Trekking a distance and long waiting time to get transportation to come to the center. ii) Getting a means of transport to access the centres. iii) Engaging in periodic checks by law enforcement officers who in some cases will ask for some form of evidence. A few had to explain to the officials that they had been exposed and had reached out to relevant institutions in which they were invited/going to take a test. With regards to the process, the respondents stated that when they got to the center, they were decontaminated/an official spoke to them and then briefed them on the processes to expect. Describing the process, the clients stated that from the entry point, they were directed to the wash station to wash their hands then they were moved to Identity verification station and the point where they filled a form from the Nigerian Centre for Disease Control, then a lab bottle was filled and the specimen collected. Also, on their way out they were sprayed again (decontaminated) before leaving the testing center and then had to wait for their results to come in via the mail.

**Symptoms, waiting phase and the testing experience:** all the respondents stated that they had experienced symptoms like cough, shortness of breath, fever, and that they had travel history and had exposure to people who were exposed to confirmed cases. Three of the respondents experienced some form of psychological distress, they were scared to take the test, they had cried and two of them found the test very invasive and painful.

**Suggestions and Recommendations:** overall, knowledge and awareness of COVID-19 are still low and there is a need to educate people, provide counsel, and increase the uptake of the testing. The majority of the clients stated that the test was scary and invasive however, the process and turnaround time was effective and prompt. They suggested that the following strategies should be explored: 1) The test is invasive and painful and other alternatives of testing should be explored. 2) Knowledge and awareness of COVID-19 is low. NIMR should educate people and create awareness at different levels. 3) Counselling should be done to reduce anxiety and distress. 4) Few people know about the centre in NIMR, awareness could increase the number of persons tested.

## Discussion

The study used a mixed-method approach to evaluate the modified drive-through sampling strategy for sample collection for the COVID-19 test. Out of the total number that visited the site, only 57.4% were found eligible of which 78.0% presented for the test. This percentage consisted of a 78.0% drive-through and a 22.0% walk-in. The average time for transiting through the drive-through site was 19.2±4.6minutes while that of the walk-in was 28±9.2min. This difference was statistically significant (p<0.001). Although 27 personnel participated in the drive-through process, a minimum of six personnel can be deployed to conduct the modified drive-through strategy per sampling cycle. During the study period, two (7.4%) out of 27 personnel tested positive for the SARS-CoV-2 infection. However, the two of them were not symptomatic. The average cost per unit participant including the cost of consumables was estimated to be 20.0USD. During the study, of the total participants that registered at the site, only 57.4% were found eligible of which, 78.0% presented for the test. This study has shown that demand creation via the internet for testing CoVid-19 could become a viable option for creating awareness in low resource settings. It also helped to screen out those who were not eligible for the test thereby reducing overcrowding at the sample collection center. This also assisted in infection control and prevention. Demand creation through the internet as in this study was used to create an alternative method for sample collection for Covid-19 test as in this project. This will invariably lead to scaling up of testing. Scaling up testing has been described by the WHO as one of the pillars for combating and containing the SARS-CoV-2 infection [10].

Interestingly, drive-through participants were more (78.0%) compared to the walk-ins (22.0%). This is contrary to what is normally observed in public institutions in Nigeria where the majority are persons who do not own cars. Cars are considered a luxury in Nigeria and most of those who own cars in Nigeria belong to the affluent group who prefer to visit tertiary hospitals that are privately owned instead of the public hospitals. Also, twenty-two per cent (22.0%) of the participants who met the eligibility criteria and were invited for the procedure could not visit the site. The possible explanation for fewer walk-ins and the marked reduction in the number of eligible participants who were invited but did not present for the test, maybe due to the possibility that they found it challenging to present due to the non-availability of public transport services. The latter was occasioned by the movement restriction measures instituted by the government. It is, therefore, possible that participants who walked to the test centre were those who lived close to the center. The average time of transit through the modified drive-through strategy was 23.6±6.9 minutes, which is twice the transit time of 10.2 minutes reported by the Korean model [4]. The longer transit time in this particular project is expected as this model includes both participants driving through and walking in. The longer transit time in this study is mainly as a result of those walking in with an average transit time of 28 minutes that was significantly longer than that of those driving in. The longer transit time for the walk-in is possibly due to the extra time spent in observing respiratory and hand hygiene practices. In this model, walk-in participants without masks were provided face masks, directed to the wash station to observe hand hygiene and then decontaminated. In contrast, only the cars were decontaminated for those who drove through.

A total of 27 persons participated in the drive-through strategy, however, a minimum of six persons were required to conduct the modified drive-through strategy per cycle. The personnel worked in shifts because of the challenges of prolonged usage of the complete set of the PPE, especially as the hours of work per day were dependent on the number of clients presenting at the center per day. Although none of the personnel developed symptoms suggestive of COVID-19, two (7.9%) members of the team who felt exposed were tested and found positive for SARS-CoV-2 infection. Following this incident, all the other team members were tested and the center closed for a period of 36 hours to allow for decontamination and retrieval of the test results. This practice is consistent with guidelines instituted by the Nigeria Centre for Disease Control [10]. No other team member tested positive to SARS-CoV-2 infection, it was, therefore, difficult to determine whether the source of the infection was from the community or at the drive-through centre. The average cost for sample collection at the drive-through centre per participant was estimated to be eight thousand naira on the average equivalent to 20 USD. This cost on surface value appears reasonable however in a resource-constrained setting like ours, with close to 60% of the citizens living on less than 2USD a day, this amount is certainly beyond the reach of many Nigerians. In this instance, most of the resources used for the testing came from donor groups and the government, hence participants did not have to pay for the test. This finding underlines the significant role that donor groups and governmental agencies play in ensuring that health services are made available to the populace who may not be able to afford the out-of-pocket payment.

In planning for the drive-through sampling modality, certain challenges anticipated include the cost of executing the project viz-a-viz sustainability and scale-up. One aspect of the drive-through sampling strategy that offers advantages over the conventional mode of sample collection is the preservation of consumables. Preservation of consumables is expected to lead to less cost as the number of PPEs required to conduct the drive-through sample collection strategy is less than the conventional method that requires a change of PPE and decontamination of the room after each procedure. On the contrary, the drive-through strategy involves a change of hand-gloves after each procedure with the preservation of the main PPE during a shift of testing. This finding was also corroborated by the model in Korea [4]. This cost-saving component of this aspect of the strategy makes it an attractive option for low- and medium-income countries where the economic challenges confronting many of these countries have made them seek for cost-saving and safe options to scale up COVID-19 testing. All the participants involved with the in-depth interview were satisfied with the services received from the Nigerian Institute of Medical Research (NIMR). They offered ratings of between eighty per cent and ninety-nine-point nine per cent. A participant stated, ‘The NIMR staff are very professional, right from the gate our car was disinfected, we were given face masks, information about the test was provided and the test was done promptly’. Another participant stated that ‘I was scared to take the test but when I came to NIMR, they spoke to me and made me feel comfortable and relaxed, also I spoke to a counselor who reassured me of the process’ The implication of this is that personnel attitude in the setting of healthcare delivery plays a crucial role in client satisfaction. Client satisfaction in the setting of healthcare delivery has been described as a fundamental factor in the determination of success in any healthcare program [11].

Another important finding from this study is the role of counselling before and after taking a test as one of the clients who was apprehensive about the test became reassured after speaking to a counsellor. Concerns have been raised about the psychological well-being and mental health of people presumed to have COVID-19 as Xiang et al. emphasized the need to cater for the psychological well-being of patients being evaluated for COVID-19 [12]. In our environment, myth and cultural beliefs tend to accentuate anxiety and perception towards undertaking a test. This is particularly true for a novel disease like CoVoid-19 that has been highly influenced by myths, controversies, and conspiratory theories. There is, therefore, a need to integrate formal counselling before and after conducting the test as seen in testing for some other disease conditions such as HIV where there is pre and post-test counselling. The health workers indicated satisfaction with the process of conducting the test. They felt protected within the PPE and they all stated that the process of obtaining samples prevented unnecessary close contact with the participants. This view supports the fundamental philosophy of the drive-through strategy that is built on a foundation of minimal contact with suspected participants with COVID-19 disease. This experience of the health workers mirrors the pattern established in the Korean model of the drive-through strategy that was stated to be a huge success [4].

## Conclusion

The test strategy although largely successful, is largely dependent on Internet penetrability. In low- and medium-income countries where internet penetration is not yet optimal, establishing this test strategy as the sole modality of testing may disenfranchise a large proportion of the populace from participating in the test. Other limitations include the possibility of inadvertently promoting inequity in sample collection in the community and, the fact that only those who have cars are likely to present at the drive-through platform despite the introduction of a walk-in component. Only 22% of participants were in the walk-in group. These limitations, therefore, mean that this testing modality will be best utilized as an adjunct to the conventional mode of testing and not as a substitute.

**What is known about this topic**

- Crucial to the global effort to ramp up strategies to contain and defeat the novel corona virus disease (COVID-19) pandemic is the place of scaling up testing services to identify those who are already infected, track their contacts, get them tested as well as commence treatment to contain the spread of the virus;
- One of such methods involves the use of the drive-through sampling strategy. This strategy was first deployed in testing for COVID-19 in South Korea with great success and then in the United States. This method of sampling was found to be effective in early detection and diagnosis of cases and invariably greater ease of contact tracing.

**What this study adds**

- This study was aimed at evaluating the successes and challenges of implementing a modified drive-through system for SARS-CoV-2 in Nigeria;
- The goal of this modality of sample collection is to minimize the risk of transmission of infection to the health care workers, while ensuring efficient use of resources;
- Information obtained will inform policy for the scale-up of testing services in the country.

## References

[1] World Health Organization WHO Director-General’s opening remarks at the media briefing on COVID-19.

[2] Salath M, Althaus C, Neher R, Stringhini S, Hodcroft E, Fellay J (2020). COVID-19 epidemic in Switzerland: on the importance of testing, contact tracing and isolation. Swiss Med Wkly.

[3] Bryson-Cahn C, Duchin J, Makarewicz V, Kay M, Rietberg K, Napolitano N (2020). A Novel Approach for a Novel Pathogen: Using a Home Assessment Team to Evaluate Patients for 2019 Novel Coronavirus (SARS-CoV-2). Clin Infect Dis.

[4] Kwon KT, Ko JH, Shin H, Sung M, Kim JY (2020). Drive-Through Screening Center for COVID-19: a Safe and Efficient Screening System against Massive Community Outbreak. J Korean Med Sci.

[5] Tolia VM, Chan TC, Castillo EM (2020). Preliminary Results of Initial Testing for Coronavirus (COVID-19) in the Emergency Department. West J Emerg Med.

[6] Jason B (2020). How South Korea reined in the outbreak without shutting everything down. Swiss Med Wkly.

[7] World Population Review Lagos Population 2020 2020.

[8] National Population Commission Nigeria Population and Housing Census.

[9] NCDC Guidelines and protocols.

[10] NCDC National Strategy to Scale-up access to Corona Virus Disease. Testing in Nigeria.

[11] Abolaji JA Patient Satisfaction with Quality Attributes of Primary Health Care Services in Nigeria.

[12] Xiang YT, Yang Y, Li W, Zhang L, Zhang Q, Cheung T (2020). Timely mental health care for the 2019 novel coronavirus outbreak is urgently needed, Lancet Psychiatry. Lancet Psychiatry.

